# Machine learning-based stratification of chagas heart failure severity using ECG power spectral biomarkers

**DOI:** 10.1007/s11517-026-03573-5

**Published:** 2026-04-15

**Authors:** Pedro Ribeiro, João Alexandre Lobo Marques, Maria Inês Barbosa, Roberto C. Pedrosa, João Paulo do Vale Madeiro, Pedro Miguel Rodrigues

**Affiliations:** 1 Universidade Católica Portuguesa, CBQF – Centro de Biotecnologia e Química Fina – Laboratório Associado, Escola Superior de Biotecnologia, Rua de Diogo Botelho 1327, 4169-005 Porto, Portugal; 2 University of Saint Joseph, Laboratory of Applied Neurosciences, Macao, 999078 China; 3 Edson Saad Heart Institute, Federal University of Rio de Janeiro, Rio de Janeiro, Brazil; 4 Federal University of Ceará, Department of Computing, Fortaleza, Ceará Brazil

**Keywords:** Chagas disease, Left ventricular ejection fraction, Power spectral density, Machine learning, Heart failure severity, Discrimination

## Abstract

**Abstract:**

**Purpose:**

This study presents a machine learning methodology to automatically classify heart failure severity in Chagas disease (CD) patients using non-invasive 24-hour ECG-Holter signals.

**Methods:**

Following American Heart Association (AHA) guidelines, the cohort was stratified into three Left Ventricular Ejection Fraction (LVEF)-based severity groups: Normal (LVEF ≥ 0.50, n=197), Moderate (0.40 ≤ LVEF < 0.50, n=106), and Severe (LVEF < 0.40, n=77), totaling N=380 patients. From short 10-second ECG segments, we extracted eleven spectral features derived from the power spectral density (PSD). Class imbalance was addressed through oversampling applied to the training folds. All classifiers were evaluated over 50 random stratified train-test splits (80/20) across three pairwise tasks (Normal vs. Moderate, Normal vs. Severe, Moderate vs. Severe).

**Results:**

Analysis revealed a consistent leftward shift in PSD, with increased low-frequency power in more severe cases, consistent with morphological ECG changes including P-wave attenuation, QRS alterations, and ST-segment shifts. Using this spectral biomarker, the best models achieved mean *AUC/PR-AUC* values of 0.79/0.76 for Normal vs. Severe and 0.83/0.85 for Moderate vs. Severe across 50 random states. The Normal vs. Moderate task showed moderate separability (*AUC* = 0.75, *PR-AUC* = 0.72).

**Conclusion:**

These findings highlight the potential of power spectral ECG analysis as a low-cost, fully automated tool for risk stratification in CD. The methodology shows promise for improving triage and clinical decision-making in resource-limited settings where CD remains highly prevalent.

**Graphical abstract:**

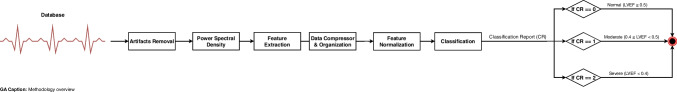

**Supplementary Information:**

The online version contains supplementary material available at 10.1007/s11517-026-03573-5.

## Introduction

Chagas disease (CD), caused by the parasite *Trypanosoma cruzi*, was first described in 1909 by Carlos Chagas. It is more prevalent in the Americas, especially in Central and South America [1, 2].

CD can manifest with both acute and chronic symptoms. The acute phase may present with fever, subcutaneous oedema, malaise, and enlargement of the spleen, liver, and lymph nodes. Additionally, some patients exhibit electrocardiogram (ECG) abnormalities such as sinus tachycardia, atrioventricular block, and T-wave changes [3].

In the chronic phase, the disease ranges from asymptomatic to highly symptomatic, with indicators grouped into cardiac, digestive, and cardiodigestive categories. Cardiac manifestations are the most frequent and consequential, affecting about 20–30% of chronic cases and causing ECG abnormalities such as right bundle branch block, left anterior fascicular block, ventricular premature beats, ST–T changes, abnormal Q waves, and low QRS voltage [3].

Diagnosis of acute CD is typically performed via microscopic detection, aiming to identify trypomastigotes in blood samples. For the chronic phase, confirmation requires at least two serological tests to detect antibodies against *Trypanosoma cruzi* [3]. Treatment during the acute phase is primarily pharmacological, with medications such as Benznidazole and Nifurtimox targeting the parasite [4]. In the chronic phase, Benznidazole remains the only available treatment in South American countries like Brazil, though no fully effective therapy exists for this stage [5].

ECG signals represent the heart’s electrical activity [6], and the characteristic changes induced by CD [7] make them a promising source of early diagnostic information [6]. Modern machine learning (ML) algorithms, originating from work in cybernetics and computer science in the 1950s [8, 9], can automatically learn patterns from such data. In this context, multi-band non-linear analysis (MLBNA) decomposes ECG signals into frequency bands and extracts non-linear features [10], providing rich ECG descriptors for ML models. Leveraging these features can accelerate detection and enhance early diagnosis and treatment of cardiovascular diseases [11].

The novelty of our approach lies in analyzing broadband spectral and power spectral ECG features extracted from 24-hour Holter data, rather than relying solely on classical Heart Rate Variability (HRV) spectral indices. Traditional HRV measures capture only narrow-band autonomic modulation and therefore overlook broader alterations in the ECG frequency structure that accompany disease progression [12]. By employing a richer and physiologically grounded representation of the signal, our framework enables the characterization of a shift in spectral power from higher to lower frequencies - a pattern consistent with conduction slowing and myocardial remodeling in Chagas cardiomyopathy [13]. Such broadband and power spectral descriptors remain underexplored in the literature, despite their potential to reveal early electro-physiological signatures of cardiac dysfunction.

Building on this rationale, we extract a comprehensive set of eleven Power Spectral Density (*PSD*)-based features, including broadband spectral metrics and power ratios across the 1–25 Hz range. These descriptors are then used to train and validate ML models designed to predict Left Ventricular Ejection Fraction (LVEF) severity, a key marker of CD–induced heart failure. This work therefore presents a novel ML-based framework that leverages richer ECG-derived information to improve the non-invasive stratification of CD-related cardiac dysfunction.

## Literature review

To establish a comprehensive overview of the state-of-the-art, a systematic literature review was conducted from August to October 2025, with Google Scholar serving as the primary search engine. This review focused on scholarly articles from prominent publishers (i.e., Elsevier, MDPI, Springer Nature, Wiley, SAGE, Taylor & Francis, and PLOS), as well as conference proceedings from SBIS and CinC, and publications from the Al-Furat Al-Awsat Technical University. The search covered the period from 2018 to the present to ensure the inclusion of recent and relevant advances in the field. A set of specific keywords guided the search: “Chagas Disease classification," “Chagas disease Deep learning," “Chagas disease Ejection fraction classification," “Chagas Disease ECG screening," “Left ventricular ejection fraction machine learning," and “Cardiovascular diseases LVEF classification." Table 1 summarizes recent state-of-the-art approaches (2019–2024) for heart failure diagnosis from CD or other cardiac conditions based on LVEF severity. The review includes 12 key LVEF-focused studies, 4 of which specifically address CD, illustrating substantial progress in ML for this domain. Methods range from conventional algorithms (e.g., Random Forest) to deep learning architectures (CNNs, ANNs, ResNets). Reported accuracies span 74.60%–92.41%, with 9 studies achieving an Area Under the Receiver Operating Characteristic Curve (*AUC*) > 0.80. The best performance, by Alkhodari et al. [14], reached 92.41% *accuracy* and *AUC* = 0.975 using a deep learning classifier on clinical features.

**Table 1:** Summary of state-of-the-art studies on diagnosing heart failure cardiac conditions deverived from CD or other diseases. Each entry details the database, comparison groups, features, classifier, limitations, and accuracy

Ref	Year	Features Extracted	Comparison Group (number of samples)	Classifier	Limitation	Validation	*Acc*	*AUC*
LVEF derived								
from CD								
Brito, B. et. al. [15]	2021	Features extracted from neural network	LVSD (93) vs. No-LVSD (1211)	AI-ECG	Unbalanced dataset.	Hold-out	89%	0.874
Silva, L. et. al. [16]	2021	28 HRV features	Preserved (38) vs. Reduced (15) vs. Borderline (10)	Random Forest	Small and unbalanced dataset.	Cross-validation	74.60%	-
Ferreira, G. et. al. [17]	2023	Features extracted from ANN	not preserved LVEF (17) vs. preserved LVEF (16)	ANN	Small test set.	Hold-out	79%	-
Madeiro, J. et. al. [18]	2023	Statistical measures related to waveform amplitudes and durations, wavelet decomposition coefficients, heart rate variability parameters and non-linear analysis	LVEF (19) vs. non-LVEF (36)	GBC	Unbalanced dataset.	Hold-out	-	0.75
LVEF derived from								
other diseases								
Attia, Z.et. al. [19]	2019	Features extracted from CNN	ALVD (4131) vs. non-ALVD (48739)	CNN	Unbalanced dataset.	Hold-out	85.7%	0.93
Kwon, J. et. al. [20]	2019	Sex, rhythm, age, weight, heart rate and QT interval	HFrEF (5245) vs. no-HFrEF (5245)	DEHF	They included patients with multiple ECGs; this could slightly bias survival analysis since multiple entries per individual.	Hold-out	-	0.889
Alkhodari, M. et. al. [14]	2021	Age, BMI, Diabetes, Anti-arrhythmics, Diuretics, VT, prior MI	HFpEF (129) vs. HFnEF (92) vs. HFrEF (82)	CNN	Small dataset.	Cross-validation	92.41%	0.975
Vaid, A. et. al. [21]	2022	Features extracted from CNN	LVEF (17840) vs. non-LVEF (129201)	CNN	Unbalanced dataset.	Hold-out	-	0.94
Choi, J. et. al. [22]	2022	features extracted from CNN	HFrEF (600) vs. No-HFrEF (691)	DeepECG-HFrEF	They included patients with multiple ECGs; this could slightly bias survival analysis since multiple entries per individual.	Hold-out	-	0.844
Akerman, A. et. al. [23]	2023	Features extracted from 3D-CNN	HFpEF (646) vs. non-HFpEF (638)	3D-CNN	Possibility that some control participants had subclinical diseases. Complete matching for age was not possible where patients with HFpEF were older.	Hold-out	-	0.95
Decoodt, P. et. al. [24]	2024	Features extracted from AutoML	rEF (208) vs. mEF (64) vs. pEF (143)	AutoML	Unbalanced dataset.	Hold-out	75.9%	0.856
Younis, S. et. al. [25]	2024	TP (ms), QT (ms), ST-T (ms), QRS (ms), Mean-Entropy and Mean-Instant requency	HFmEF (92) vs. HFpEF (129) vs. HfrEF (82)	Decision Trees	Small dataset.	Cross-validation	91.2%	0.98

Analysis of Table 1 shows substantial variation in dataset sizes and validation strategies. Sample sizes range from 33 subjects in Ferreira et al. [17] to over 147000 in Vaid et al. [21]. This disparity is critical, as 9 of the 12 studies explicitly report limitations due to small or unbalanced datasets, highlighting a persistent challenge for generalizable models. Validation methods also vary: 9 studies used hold-out validation, whereas only 3 employed cross-validation. This predominance of hold-out validation, especially in smaller cohorts, suggests that future work should adopt more rigorous validation protocols to improve reliability and reproducibility.

## Methodology and materials

**Fig. 1:**
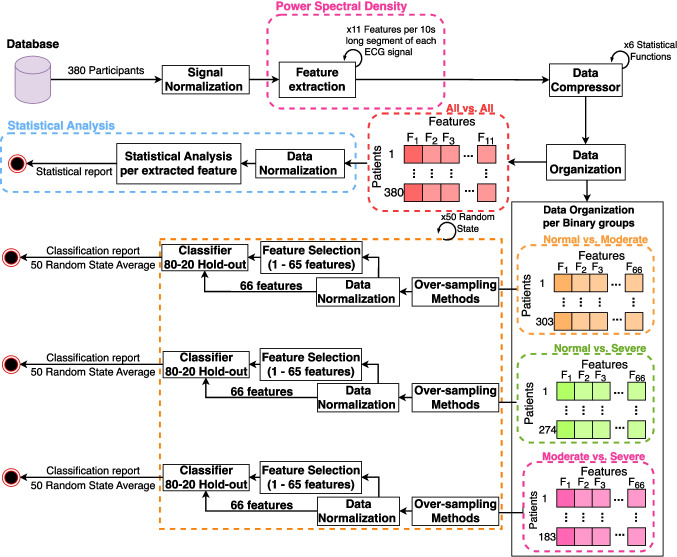
Methodology workflow diagram

Our methodology (Fig. 1) comprised ECG acquisition, automated artifact detection, normalization, and feature extraction. After extracting the eleven *PSD*-based features, the dataset was evaluated across 50 random train–test splits (80/20). The entire preprocessing pipeline, including automated oversampling, feature selection, and classifier training, was fitted exclusively on the training split for each iteration, ensuring no information leakage. Five machine learning classifiers were then evaluated on the independent test sets using metrics such as *AUC*, Precision–Recall AUC (*PR-AUC*), *Accuracy*, *Recall*, and *Precision*.

### The dataset

#### Population characterization

The data originate from a cross-sectional, descriptive study involving 380 patients (56% female) treated for Chagas Heart Disease (ChHD) at Hospital Universitário Clementino Fraga Filho (HUCFF) and the Federal University of Rio de Janeiro (UFRJ) between 1992 and 2016. All patients were managed according to the protocols of the Brazilian Ministry of Health [26].

From the initial cohort of 556 individuals, strict exclusion criteria were applied to ensure that the final study population represented a clinically homogeneous group with *confirmed* Chagas cardiomyopathy. A total of 82 patients were excluded due to malignancy, psychiatric disorders, severe hepatic disease (cirrhosis), advanced renal failure (dialysis or renal transplantation), or other forms of non-cardiac organ failure. Sixteen patients were excluded due to loss to follow-up, 17 were removed because they had received anti-*T. cruzi* treatment (based on disease duration, absence of severe cardiomyopathy, and patient preference), and 7 were excluded due to having undergone isolated cardiac resynchronization therapy. After these exclusions, 434 eligible patients were invited to participate in a structured clinical follow-up programme. At enrollment, additional exclusion criteria were applied: atrial fibrillation, anomalies in ECG analogue-to-digital conversion, or the presence of a ventricular-paced rhythm. This resulted in a final cohort of 380 patients. Importantly, all patients included in the final dataset had a definitive diagnosis of Chagas cardiomyopathy, established by at least two positive serological tests for *T. cruzi* infection together with compatible electrocardiographic abnormalities, following established diagnostic criteria. No patients with other etiologies of cardiomyopathy or alternative cardiac pathologies were included.

#### Ethical approval

Data acquisition and usage were approved by the institutional ethics committee (approval no. 45360915.1.1001.5262) of HUCFF. In accordance with the Declaration of Helsinki and Brazilian research ethics standards, the requirement for written informed consent was waived.

#### Data acquisition and target variable

Each patient record included a 24-hour ECG Holter (Lead II) acquisition sampled at 128Hz. The LVEF, calculated using the Teicholz method, served as the target variable for classification. All ECG Holter recordings included in this study were obtained exclusively from patients with confirmed chronic Chagas cardiomyopathy, as ensured by at least two positive serological tests for T. cruzi antibodies together with characteristic ECG abnormalities. This guaranteed that no signals from non-Chagas etiologies were inadvertently incorporated into the dataset. The 24-hour Holter recordings and the echocardiographic examinations used to estimate LVEF were performed with a median interval of 6.3 months (interquartile range: 3.2–8.9 months). Both assessments were systematically conducted between 8:00 a.m. and 12:00 p.m., a time window characterized by heightened sympathetic activity [27, 28], thereby maximizing autonomic modulation of cardiac electrophysiology. Harmonizing the timing of these examinations minimized circadian physiological variability and strengthened the temporal alignment between ECG-derived features and the corresponding LVEF measurements. During Holter monitoring, patients documented symptoms, activity levels, and relevant daily events to contextualize physiologic fluctuations observed in the raw signal. Thus, all computational analyses performed on present work relied exclusively on the previously mentioned 4-hour segment, selected to optimize signal quality and minimize artifacts. All remaining portions of the 24-hour ECG were excluded to ensure methodological uniformity across participants.

Echocardiographic measurements followed the recommendations of the American Society of Echocardiography. Although LVEF was calculated using the Teicholz M-mode method, an additional subjective visual assessment by experienced cardiologists was incorporated, given that regional wall-motion abnormalities and segmental asynergia, common in chronic Chagas cardiomyopathy, may impair formula-based LVEF estimation accuracy. Visual assessment has been shown to outperform automated estimation under such heterogeneous contraction patterns [29]. All echocardiographic studies were recorded and independently reviewed by two observers to ensure interpretative consistency. Likewise, Holter recordings were analyzed twice by the same observer (at acquisition and during dataset preparation) to ensure consistent annotation, artifact detection, and signal-quality verification across the dataset.

Following the American Heart Association (AHA) guidelines [30–33], the dataset was stratified into three heart-failure severity groups based on LVEF: Normal (LVEF $\ge 0.50$, 197 samples), Moderate ($0.40 \le \textrm{LVEF} < 0.50$, 106 samples), and Severe ($\textrm{LVEF} < 0.40$, 77 samples). These categories were consistently applied throughout the study, and a detailed characterization of the cohort is provided in Table 2. ANOVA analyses revealed that only LVEF differed significantly across the three groups ($p<0.05$), with all pairwise post-hoc comparisons confirming robust between-group differences, whereas Sex, BMI, and Age showed no statistically significant variation across groups.

**Table 2:** Demographic and clinical characteristics of the study population (Mean ± SD), presented for the full cohort and stratified by LVEF-based classes. F = female; M = male

	eneral		Normal		Moderate		Severe	
Sex	220 F	160 M	110 F	87 M	62 F	44 M	49 F	28 M
Age	44.55 ± 9.30	43.59 ± 9.86	44.21 ± 9.14	43.34 ± 9.67	45.43 ± 9.19	40.20 ± 9.88	44.18 ± 9.91	46.71 ± 9.20
BMI	29.15 ± 13.45	27.80 ± 2.86	29.75 ± 19.02	28.18 ± 3.08	28.81 ± 2.58	27.51 ± 2.56	28.24 ± 2.31	27.07 ± 2.45
LVEF	0.52 ± 0.16		0.66 ± 0.07		0.44 ± 0.02		0.31 ± 0.07	

### Experimental setup

All computational tasks were executed on a MacBook Pro (14-inch, 2021) equipped with an Apple M1 Pro system-on-a-chip (8-core CPU, 14-core GPU) and 16 GB of unified memory. The software tool-chain consisted of Matlab (R2023b, The MathWorks, Inc.) for spectral feature extraction, signal preprocessing, and data organization, while Python (3.9.12, Python Software Foundation) was used for the statistical analysis and reporting & evaluation of ML model performance.

### Signal filtering

Each ECG signal was filtered using a 16th-order elliptic band-pass filter with a pass-band of 1–25 Hz (steepness = 0.85; stop-band attenuation = 60 dB). This upper cutoff frequency was selected after re-examining the spectral content of our recordings, which confirmed that the relevant physiological power of the ECG is concentrated below 25 Hz. Defining the filter in this range ensures that all meaningful ECG components are preserved while higher-frequency noise is effectively suppressed. This choice is further supported by recent findings in the literature. Ádám et al. (2025) [34] demonstrated that an upper frequency boundary around 20–25 Hz captures all important ECG signal characteristics, with no relevant information being lost. This evidence reinforces the adoption of a 25 Hz upper cutoff in our processing pipeline.

### Artifact removal

**Fig. 2:**
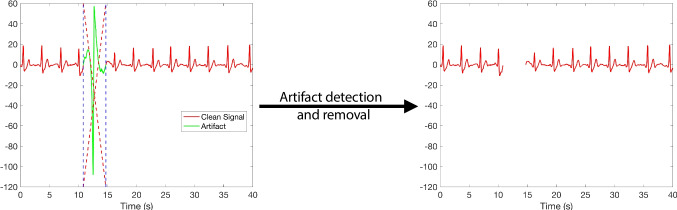
40-seconds of ECG signal’s example for artifact detection and removal

To ensure signal quality, a custom artifact removal algorithm was developed, as outlined in Algorithm on Table B1 at Supplementary Material B to remove artifact of the same nature as the one in the Fig. 2. To establish a robust baseline for artifact detection, the median value of each previously filtered signals is computed. The core of the algorithm involves identifying all local peaks in the signal and comparing their amplitudes against dynamic thresholds derived from the median. Any peak with an amplitude exceeding 50 times the median (indicating a large spike artifact) or falling below 0.015 times the median (suggesting noise or a baseline distortion) is classified as an artifact and their located zone (the time-series between the previously peak and the identified peak as an artifact) is removed from the signal. The peaks are processed in descending order of amplitude to ensure that the largest artifacts are removed first.

After removing artifacts from the ECG recordings, we analyzed the remaining signal segments for each subject. Rather than reconstructing a continuous signal, which could introduce artificial information or bias, we performed all analyses directly on these segments using a sliding-window approach. To ensure the robustness of the procedure, we discarded all segments shorter than 10 s, retaining only those with a duration exceeding 10 s for subsequent analysis. As a direct consequence of this quality-driven segmentation, the total amount of analyzable data inherently varies across subjects, depending on the number and duration of artifact-free segments preserved. After preprocessing, the mean SNR of the recordings was 12 dB, which falls within the typical range reported for long-term ambulatory ECG monitoring (5–15 dB) [35]. This confirms that the cleaned signals preserved sufficient quality for reliable spectral analysis.

### Signal normalization

After artifact removal, the ECG filtered signals, *x*(*n*), were normalized according to the Root Mean Square (RMS) normalization formula:

1 $$\begin{aligned} x(n) = \frac{x(n)}{\sqrt{\frac{\sum _{n=0}^{N-1}x^2(n)}{N}}}, \end{aligned}$$

where *N* is the ECG length. Then, the mean value was removed from the entire signal.

### ECG time-frequency analysis through power spectral density and feature extraction,

Spectral analysis is a fundamental technique for interpreting ECG signals, typically involving the decomposition of the time-domain signal into its constituent frequency components [36]. For stationary signals, this transformation is commonly achieved using the Fourier Transform (FT), which extracts spectral information by projecting the signal onto a basis of sinusoidal functions.

Given an ECG signal $x(t)$ sampled at a frequency $f_s = 1/T_s$, where $T_s$ is the sampling period, the resulting discrete-time signal is $x[n] = x(n T_s)$ for $n = 0, \ldots , N-1$, where $N$ is the total number of samples. The spectrum of this signal, $X[k]$, can be obtained using the Discrete-Time Fourier Transform (DTFT). The DTFT assumes that $x[n]$ is periodic with a fundamental period $N$, corresponding to a fundamental frequency of $\Omega _0 = 2\pi /N$. The discrete frequency variable $k$ then corresponds to harmonics of this fundamental frequency, such that $\Omega = k\Omega _0$.

The forward and inverse DTFT are defined by the transform pair:

2 $$\begin{aligned} X[k]&= \sum _{n=0}^{N-1} x[n] \cdot e^{-j k \Omega _0 n}, \quad k = 0, \ldots , N - 1 \end{aligned}$$

3 $$\begin{aligned} x[n]&= \frac{1}{N} \sum _{k=0}^{N-1} X[k] \cdot e^{j k \Omega _0 n}, \quad n = 0, \ldots , N - 1 \end{aligned}$$

where $X[k]$ are the complex Fourier coefficients. This transform pair provides a complete representation of the signal in the frequency domain, allowing for lossless recovery of the time-domain signal from its spectral components and vice-versa [37, 38].

The distribution of signal power across different frequencies is a key characteristic, formally described by the *PSD*. For a discrete-time signal, a common method for estimating the *PSD* is via the Wiener-Khinchin theorem, which states that the *PSD* is the Fourier transform of the signal’s autocorrelation function [39]. The unbiased autocorrelation estimate, $R_{xx}[u]$, for a lag $u$ is given by:

4 $$\begin{aligned} R_{xx}[u] = \left\{ \begin{array}{ll} \frac{1}{N} \sum _{n=0}^{N-m-1} x[n] \cdot x^*[n+u], & \text {if } u \ge 0 \\ R^*_{xx}[-u], & \text {if } u < 0 \end{array}\right. \end{aligned}$$

where $x^*$ denotes the complex conjugate (for real signals, $x^* = x$). The *PSD* is then estimated as the DTFT of this autocorrelation sequence:

5 $$\begin{aligned} PSD[k] = \frac{1}{N} \sum _{u=0}^{N-1} R_{xx}[u] \cdot e^{-j \frac{2 \cdot \pi k}{N-1}}, k = 0, \ldots , N-1 \end{aligned}$$

To facilitate the computation of spectral features, the *PSD* was normalized. Specifically, each *PSD* was divided by its total power (the integral across all frequencies), thereby scaling the total spectral content to unity (1).

6 $$\begin{aligned} PSD_n[k]=\frac{PSD[k]}{ \sum _{k=0}^{N_T-1} PSD[k]} \end{aligned}$$

7 $$\begin{aligned} PSD_n(f)=PSD_n \left[ \frac{k*f_s}{N_T}\right] \end{aligned}$$

where $N_T$ is the length of the $PSD_n$ and *f* is the frequency bins.

**Fig. 3:**
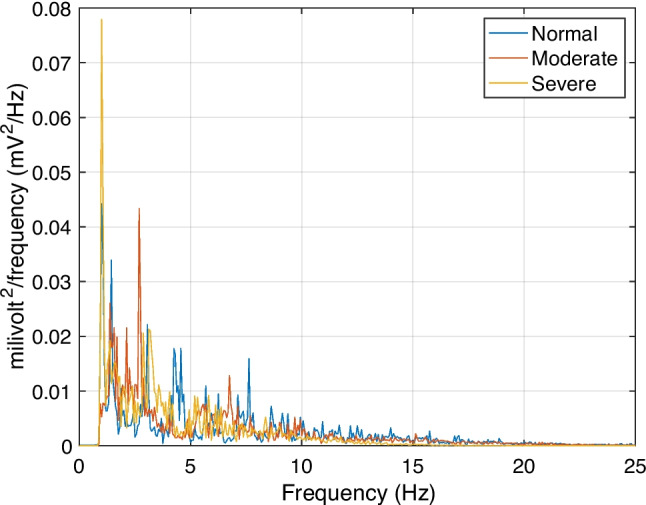
*PSD* plot for the 3 classes

Figure 3 shows the *PSD* for the 3 classes, while Fig. C1 at Supplementary Material C includes the relative power percentages for each plot, A noticeable trend in the latter is the leftward shift of the power spectra, indicating that spectral power concentrates at lower frequencies as the disease develops.

### Feature extraction

With the spectral “lentification” observed in Fig. C1 in Supplementary Material C, and because this phenomenon is fundamentally expressed as changes in *relative* spectral power rather than absolute amplitude, ratio-based and power-derived metrics provide a sensitive and physiologically meaningful way to capture these shifts.

In the first stage of a two-stage feature extraction process, we followed the standard duration of ECG recordings in clinical practice [40] and applied a 10-second, non-overlapping sliding window to the clean segments to compute their *PSD*. From each *PSD* we extracted eleven *PSD*-based features, summarized in Table 3.

**Table 3:** Features extracted, corresponding equations, and short description

Feature	Equation	Description
Median Frequency (*MF*)	$MF = f_i \ \text {such that} \ \frac{\sum _{1 \le f \le f_i} PSD(f)}{\sum _{1 \le f \le 25} PSD(f)} = 0.5$	Frequency at which 50% of the PSD within 1–25 Hz is accumulated.
Relative Power of low Frequencies (*RP*1)	$RP1 = \sum _{1Hz}^{6.25Hz} PSD_n(f)$	Relative power in the low-frequency band (1–6.25 Hz).
Relative Power of intermediate Frequencies (*RP*2)	$RP2 = \sum _{6.25Hz}^{12.5Hz} PSD_n(f)$	Relative power in the intermediate band (6.25–12.5 Hz).
Relative Power of intermediate high Frequencies (*RP*3)	$RP3 = \sum _{12.5Hz}^{18.75Hz} PSD_n(f)$	Relative power in the mid–high band (12.5–18.75 Hz).
Relative Power of high Frequencies (*RP*4)	$RP4 = \sum _{18.75Hz}^{25Hz} PSD_n(f)$	Relative power in the high-frequency band (18.75–25 Hz).
Ratio between *RP*1 and *RP*2 ($R_1$)	$R_1 = \frac{RP1}{RP2}$	Ratio of low- to intermediate-frequency power. Higher values indicate dominance of low-frequency activity.
Ratio between *RP*1 and *RP*3 ($R_2$)	$R_2 = \frac{RP1}{RP3}$	Ratio of low- to mid–high-frequency power.
Ratio between *RP*1 and *RP*4 ($R_3$)	$R_3 = \frac{RP1}{RP4}$	Ratio of low- to high-frequency power.
Ratio between *RP*2 and *RP*3 ($R_4$)	$R_4 = \frac{RP2}{RP3}$	Ratio of intermediate to mid–high frequency power.
Ratio between *RP*2 and *RP*4 ($R_5$)	$R_5 = \frac{RP2}{RP4}$	Ratio of intermediate to high-frequency power.
Ratio between *RP*3 and *RP*4 ($R_6$)	$R_6 = \frac{RP3}{RP4}$	Ratio of mid–high to high-frequency power.

To compute these related power features (relative power and ratios), the 1–25 Hz physiological band was divided into four equal-width sub-bands. This uniform segmentation provides a balanced and unbiased partitioning of the spectrum, ensuring that the *PSD* is represented consistently across its entire physiologically relevant range. Importantly, equal-width sub-bands are particularly well suited to characterizing the progressive left-shift in spectral power observed with advancing disease: as energy becomes increasingly concentrated at lower frequencies, the symmetric partition allows this redistribution to be quantified without imposing a priory assumptions about which regions should dominate. This yields descriptors that are mathematically stable and physiologically interpretable, capturing disease-related changes in the structure of the ECG spectrum.

For each of the eleven time series, we computed six summary statistics (stats): the mean, median, standard deviation (SD), variance (Var), 95th percentile (P95), and kurtosis (Kur) [41]. These summary measures are hereafter denoted by the configuration feature_stat. This resulted in a final set of 66 features (11 primary features $\times$ 6 statistical metrics) for each patient record, totaling 380 patients.

### Feature normalization

Prior to model training, all 66 features were standardized using z-score normalization, where each value $x_i$ - where $1 \le i \le 66$ - is transformed to $\tilde{x}_i$ according to:

8 $$\begin{aligned} \tilde{x}_i = \frac{x_i - \mu }{\sigma } \end{aligned}$$

To prevent data leakage and ensure analytical validity, the normalization parameters (mean ($\mu$) and standard deviation ($\sigma$)) were computed from the training data and then applied to both the training set and the test set (data splitting explained in the following point Section 3.10). For pairwise comparisons, feature normalization used a reference population comprising only the two groups being analyzed, whereas for all-versus-all comparisons, a pooled reference population from all groups was used.

### Statistical analyses

To assess differences among the Normal, Moderate, and Severe groups, a statistical analysis was conducted on the mean values of the eleven primary features. The assumption of normality for each feature was evaluated using the Kolmogorov–Smirnov test, and homogeneity of variances across the three groups was assessed using Levene’s test. Once these assumptions were confirmed, a one-way Analysis of Variance (ANOVA) was performed to test for significant differences among the three groups, with statistical significance defined as $p < 0.05$; post-hoc pairwise comparisons were also performed between the groups. To control for multiple comparisons across these pairwise tests, the Bonferroni correction was applied.

### Combined features power to discriminate between groups via ML approaches

To evaluate pairwise classification performance across study groups, we employed five ML models from scikit-learn [42], detailed in Table 4, using a repeating stratified 80/20 split across 50 different random seeds, effectively emulating the robustness of repeated cross-validation, as the model is evaluated on 50 independent test sets.

**Table 4:** Configurations of the five Scikit-learn classifiers used in this study

Classifier (Scikit-learn class)	Hyperparameters
AdaBoost (AdaBoostClassifier)	(n_estimators = 50, learning_rate = 1.0, algorithm = “SAMME.R”) + default
Decision Tree (DecisionTreeClassifier)	(max_depth = 5, criterion = “gini”, splitter = “best”, min_samples_split = 2) + default
K-Nearest Neighbors (KNeighborsClassifier)	(n_neighbors = 5, weights = “uniform”, algorithm = “auto”) + default
Linear SVM (LinearSVC)	(penalty = “l2”, loss = “squared_hinge”, dual = True) + default
RBF SVM (SVC)	($\gamma$ = “auto”, kernel = “rbf”, C = 1.0, probability = False) + default

A comprehensive preprocessing and training pipeline was fitted exclusively on the training data, ensuring a robust evaluation fully insulated from data leakage. This entire procedure was executed independently for each random seed, and the pipeline consisted of three sequential stages:

1. **Class Balancing:** OS techniques (specifically, RandomOverSampler - ROS and ADASYN) were applied to the features to mitigate the effects of class imbalance in the training data.
2. **Feature Normalization:** Following class balancing, we applied z-score normalization to all features’ time-series, as previously described in the Methods Section 3.8.
3. **Feature Selection:** With the features normalized, the next step was to identify the optimal feature subset for each classification task, we employed an ANOVA F-test. Features were ranked by their F-value, which measures the ratio of between-group to within-group variance and indicates discriminatory power [43]. This ranking was used to evaluate subsets of increasing size, from 1 to 66 features, to find the optimal set.
4. **Model Training:** Finally, each classifier was trained on the transformed (feature-selected and balanced) training data.

The fully trained model was then evaluated on an independent test sets across 50 random-state runs, using data that was never involved in feature selection or oversampling procedures. All reported performance metrics correspond to the average across these 50 runs, ensuring a stable and robust estimate of model performance.

#### Classification evaluation metrics

Model performance was evaluated using a suite of standard classification metrics, with the *AUC* as the primary indicator [44]. To better contextualize the model’s clinical applicability, particularly in scenarios where sensitivity-driven triage may be relevant, we also added the *PR-AUC*. The *AUC* and *PR-AUC* summarize a model’s ability to discriminate between classes across all thresholds, making it effective for comparing models, particularly on imbalanced datasets. For a more granular analysis, we also computed Accuracy (*Acc*), Recall (*Rec*), Specificity (*Spec*), Precision (*Prec*), Negative Predictive Value (*NPV*), and the F1-Score (*F1*) [45] (more information at Supplementary Material A).

## Results and discussion

This section details the outcomes of our study, beginning with the statistical analysis of the extracted features and culminating in the performance of the ML classifiers.

### Separability between groups, a multiclass and pairwise evaluation via statistical analysis

To investigate the statistical separability between classes, we employed an ANOVA across all 3 groups, followed by pairwise t-tests. The overall ANOVA identified 15 feature distributions with statistically significant differences (Supplementary Material C - Fig. C2), with the most significant result (*p*=0.004) associated with the $R_{1}$ feature using the *Kur* data compressor.

Drilling down into pairwise comparisons revealed where these differences were most pronounced. The distinction between *Normal* and *Severe* classes was the strongest, yielding 15 significant feature distributions, with a top *p*-value of 0.0005 from the $R_{1}$ feature (*Kur* compressor) (Figure in Fig. C4 in Supplementary Material C). The *Moderate* vs. *Severe* separation was also robust, with 11 significant distributions and a best *p*-value of 0.0043, from the *RP*4 feature (95*Prc* compressor) (Supplementary Material C - Fig. C5). In stark contrast, the *Normal* vs. *Moderate* comparison comparison did not produce distributions with significant differences (Fig. C3 in Supplementary Material C). This confirms that while the classes are separable overall, the distinction between the *Normal* and *Moderate* groups is the most subtle and challenging.

### Spectral power shift evaluation

Table 5 summarizes the mean values of the features across the three patient classes. As set on point 3.9., a one-way ANOVA was performed to assess spectral-power differences between classes by testing the extracted features stratified by patient class based on their mean values, revealing that five of the eleven primary features (45.45%) differed significantly across groups.

**Table 5:** Mean ± SD distribution of primary features stratified by patient class. Group differences were evaluated using one-way ANOVA with Bonferroni-adjusted post-hoc tests, and independent-samples t-tests when multiple-comparison differences were significant. *N*.*S*. indicates $p \ge 0.05$. Features correspond to MF, RP1–RP4, and their ratios $R_1$–$R_6$

Feature	*p*-value	Mean ± SD (Normal, 197 samples)	Mean ± SD (Moderate, 106 samples)	Mean ± SD (Severe, 77 samples)	*p*-value Normal vs. Moderate	*p*-value Normal vs. Severe	*p*-value Moderate vs. Severe
MF_Mean	N.S.	9.93 ± 3.60	9.99 ± 3.26	8.81 ± 3.08	N.S.	0.017	0.014
RP1_Mean	0.022	0.28 ± 0.18	0.27 ± 0.17	0.35 ± 0.19	N.S.	0.0047	0.0053
RP2_Mean	0.0376	0.22 ± 0.09	0.23 ± 0.08	0.26 ± 0.09	N.S.	0.0036	0.037
RP3_Mean	N.S.	0.15 ± 0.06	0.16 ± 0.06	0.17 ± 0.06	N.S.	0.039	N.S
RP4_Mean	0.0191	0.12 ± 0.05	0.13 ± 0.05	0.10 ± 0.06	N.S.	0.0046	0.0051
R$_1$_Mean	N.S.	1.44 ± 1.14	1.41 ± 1.05	1.61 ± 1.34	N.S.	N.S.	N.S.
R$_2$_Mean	N.S.	2.72 ± 3.32	2.51 ± 2.85	3.64 ± 6.58	N.S.	N.S.	N.S.
R$_3$_Mean	N.S.	5.40 ± 9.26	7.80 ± 26.63	38.01 ± 241.6	N.S.	N.S.	N.S.
R$_4$_Mean	N.S.	1.82 ± 1.31	1.91 ± 1.70	1.97 ± 1.45	N.S.	N.S.	N.S.
R$_5$_Mean	0.0329	3.12 ± 4.62	3.95 ± 9.70	8.73 ± 27.97	N.S.	0.0068	N.S.
R$_6$_Mean	0.0051	1.68 ± 1.60	1.79 ± 1.78	2.9 ± 4.71	N.S.	0.0015	0.027

Measures indexing spectral location and power showed the strongest associations with disease severity. In particular, the mean relative power in the lower frequency band (RP1_Mean) increased with severity, rising from 0.28 ± 0.18 (Normal) and 0.27 ± 0.17 (Moderate) to 0.35 ± 0.19 (Severe), reflecting a significant shift of spectral power toward lower frequencies (*p* = 0.022). Similarly, the mean relative power in the intermediate frequency band (RP2_Mean) increased from 0.22 ± 0.09 (Normal) and 0.23 ± 0.08 (Moderate) to 0.26 ± 0.09 (Severe), also indicating a significant redistribution of spectral power toward lower frequencies (*p* = 0.0376). In contrast, the mean relative power in the high-frequency band (RP4_Mean) decreased with severity, from 0.12 ± 0.05 (Normal) and 0.13 ± 0.05 (Moderate) to 0.10 ± 0.06 (Severe), further supporting a significant shift of spectral power toward lower frequencies (*p* = 0.0191). Finally, it should be emphasized that, although a slight trend toward lower-frequency displacement shown by some features is observable between the Normal and Moderate groups, these differences did not reach statistical significance (Bonferroni-corrected $p \ge 0.05$), indicating that the spectral shift becomes substantial only from Moderate to Severe. This leftward spectral shift is corroborated by the *PSD* plots (Fig. C2 in Supplementary Material C) and interestingly, while our study is in a different field, these findings are coincident with previous reports of spectral slowing in other domains such as neuro-degenerative conditions [46–52]. We examined the ECG morphologies to understand the leftward shift in power observed in the spectral features. As illustrated in Fig. C7 in Supplementary Material C, the class-specific alterations visible in the ECG traces help explain this behavior: (i) in the Moderate class, reductions in P-wave amplitude, occasional QRS-complex changes and mild ST-segment elevation reduce high-frequency content; (ii) in the Severe class, the absence of a clear P wave and a markedly elevated ST segment further attenuate higher-frequency components; and (iii) these progressive morphological degradations smooth the waveform and diminish abrupt transitions, shifting energy toward lower frequencies and reinforcing the leftward spectral shift.

### Classification performance

Following the feature analysis, we evaluated the performance of 5 classifiers on the 3 binary classification tasks. The criteria to select the best results per comparison group was based on the best *PR-AUC*. The optimal results for each task are summarized in Table 6.

**Table 6:** Optimal mean classification results for each pairwise comparison across 50 random states, indicating the best-performing model and its associated metrics

Pairwise	OS	Classifier	*Acc*	*Rec*	*Prec*	*Spec*	*F1*	*NPV*	*AUC*	*PR-AUC*
Normal vs. Moderate	ROS	DecisionTreeClassifier	77.05	82.50	82.50	66.67	82.50	66.67	0.7458	0.724
Normal vs. Severe	ROS	SVC	83.64	89.74	87.50	68.75	88.61	73.33	0.7925	0.7559
Moderate vs. Severe	ROS	KNeighborsClassifier	83.78	85.71	85.71	81.25	85.71	81.25	0.8348	0.853

*Acc* = Accuracy; *AUC* = Area Under the Curve; *F*1 = *F*1-score; *NPV* = Negative Predictive Value; OS = oversampling; *Prec* = Precision; *Rec* = Recall; *Spec* = Specificity; $PR-AUC$ = Precision–Recall AUC

The classification task distinguishing Moderate from Severe achieved the highest performance, with the KNN classifier reaching an *AUC* of 0.8348, a *PR-AUC* of 0.853, and an *Acc* of 83.78%. The Normal vs. Severe comparison also showed strong discriminative ability, with the SVC model achieving an *AUC* of 0.7925, a *PR-AUC* of 0.7559, and an *Acc* of 83.64%, although interestingly these values remain slightly lower than those obtained for Moderate vs. Severe.

In contrast, the Normal vs. Moderate classification was the most challenging task, consistent with the statistical analysis. The best-performing model (Decision Tree Classifier) achieved an *AUC* of 0.7458 and a *PR-AUC* of 0.724, indicating moderate separation between the two classes. This outcome is not unexpected, as the statistical analysis showed that Normal and Moderate differ only slightly, although some features exhibit a mild trend toward lower-frequency displacement. Nevertheless, these tendencies are insufficient to generate a clear statistical separation, which explains why the classification performance, while still showing some discriminative relevance, remains lower than in the other pairwise comparisons.

Another key finding emerged from the model optimization, the RandomOverSampler was essential for achieving top performance in all three scenarios (100%), highlighting the critical need to address class imbalance in this dataset.

Figure C6 in Supplementary Material C provides a broader view of performance, showing the distribution of PR-AUC scores across all classifiers and feature sets. The boxplots reveal three important trends: (1) performance differences among the top classifiers are relatively small, (2) the outcomes are consistent across models, offering no indication of selective reporting or model-specific artifacts, and (3) classifier choice has a comparatively smaller influence on overall performance than feature selection or data balancing strategies. Collectively, these observations strengthen the robustness and reliability of our findings.

### Comparison with the state-of-the-art

To contextualize our findings, we benchmarked our model against the state-of-the-art, as summarized in Table 1. Our analysis reveals prevalent methodological trends, including a majority of studies employing binary classifications (66.67%) and hold-out validation (75%). Furthermore, common challenges include small sample sizes (33%) and unbalanced datasets (50%). To mitigate these issues, our work directly addresses these limitations through over-sampling techniques.

Among the literature on heart failure assessed from CD with LVEF-based stratification, the studies by Ferreira et al. [17], Brito et al. [15], and Madeiro et al. [18] constitute the most directly comparable benchmarks. Compared with Ferreira et al., our model achieved a 4.64% higher accuracy in the Normal vs. Severe classification task, although it exhibited a 2% lower accuracy in the Normal vs. Moderate task. Relative to Brito et al., our approach yielded slightly lower accuracy and *AUC*; however, it should be noted that in our study we applied a different stratification scheme for the Normal and Moderate classes, considering only LVEF values above 0.4 for this separation. Most notably, our model substantially outperformed the method proposed by Madeiro et al., achieving improvements of 4.25% and 8% in *AUC* for the Normal vs. Severe and Moderate vs. Severe classifications, respectively.

When compared to studies of heart failure from other etiologies that employ similar LVEF-based class structures [20–23], our model demonstrates competitive performance. Although Kwon et al. [20], Vaid et al. [21], and Akerman et al. [23] reported higher AUC values–exceeding ours by 5.42% to 9.65% in classifications that included a “Severe” category–our model achieved a slightly lower *AUC*s relative to Choi et al. [22], with 0.92% and 5.15% less in comparable settings. Furthermore, our results offer an important point of reference for models targeting heart failure associated with CD. While our *AUC* is 11.81% below the top benchmark for this etiology, this difference must be interpreted in context: our model was trained and evaluated on a more finely stratified and balanced dataset, providing what is arguably a more robust and reliable benchmark for future research in the field.

Critically, none of the 12 papers benchmarked in Table 1 reported a similar power shift phenomenon found on present study, underscoring the novelty of our findings.

## Conclusion

We developed and validated a ML model that successfully classifies heart failure severity in CD patients using features from short ECG segments. Our results demonstrated both a statistically significant power spectral “shift" corresponding to disease severity and high classification accuracies (up to 83.78%). The primary clinical value of this approach lies in its potential as a low-cost, non-invasive tool for rapid bedside risk stratification, enabling better triage decisions in resource-limited environments [53].

To ensure model explainability, we deliberately prioritized a classical ML framework with explicit feature extraction over deep learning. This interpretable setup showed that all PSD-derived metrics were consistently modulated by the leftward shift in the power spectrum. This spectral slowing, in turn, aligns with broader morphological changes in the ECG waveform, such as reduced P-wave amplitude, alterations in QRS morphology, or shifts in ST-segment levels, providing a physiologically coherent basis for the model’s predictions.

Moving forward, research should focus on validating these findings on a larger dataset and integrating standard clinical variables to improve both generalizability and clinical acceptance. Another interesting direction would be introducing power-law *PSD*, as it showed to be useful to characterize CD [54] and compared to the results obtained in present study. Ultimately, this work provides a strong foundation for an accessible decision-support tool that can significantly aid in the management of heart failure severity induced by CD.

## Supplementary information

The online version contains supplementary material available at doi: (available after being accepted - included in submission - appendices.pdf);

## Supplementary Information

Below is the link to the electronic supplementary material.

Supplementary file 1 (pdf 1548 KB)

## Data Availability

The datasets generated during and/or analysed during the current study are available from the corresponding author upon reasonable request.
