# Supplementary material for: Machine learning-based stratification of chagas heart failure severity using ECG power spectral biomarkers
Source: Med Biol Eng Comput. 2026 Apr 15;64(6):2163–76. doi: 10.1007/s11517-026-03573-5 (PMC13269482; doi:10.1007/s11517-026-03573-5)
Supplement: Supplementary file 1 — (pdf 1548 KB) [file 11517_2026_3573_MOESM1_ESM.pdf]

Appendix A Classification Evaluation Metrics Formulas

- **Accuracy (Acc)** measures the proportion of all correctly classified instances.

$$\text{Acc} = \frac{TP + TN}{TP + TN + FP + FN} \times 100\% \tag{A1}$$

where, TP, TN, FP and FN represents the True Positives, the True Negatives, the False Positives, and the False Negatives, respectively.

- **Recall (Rec)**, or Sensitivity, measures the proportion of actual positives that were correctly identified.

$$\text{Rec} = \frac{TP}{TP + FN} \times 100\% \tag{A2}$$

- **Specificity (Spec)** measures the proportion of actual negatives that were correctly identified.

$$\text{Spec} = \frac{TN}{TN + FP} \times 100\% \tag{A3}$$

- **Precision (Prec)**, or Positive Predictive Value, measures the proportion of positive predictions that were actually correct.

$$\text{Prec} = \frac{TP}{TP + FP} \times 100\% \tag{A4}$$

- **Negative Predictive Value (NPV)** measures the proportion of negative predictions that were actually correct.

$$\text{NPV} = \frac{TN}{TN + FN} \times 100\% \tag{A5}$$

- **F1-Score** is the harmonic mean of Precision and Recall, providing a single score that balances both metrics.

$$\text{F1-Score} = 2 \times \frac{\text{Prec} \times \text{Rec}}{\text{Prec} + \text{Rec}} \times 100\% \tag{A6}$$

Appendix B Supplementary Tables

Table B1: Artifact removal pseudocode.

```
Input  : ECG filtered signal x
Output: Cleaned ECG signal z
medZ ← Median(x)
  % Calculate the Median of the filtered signal
(PKS, LOCS) ← findpeaks(|z|)
  % Gets the peak value (PKS) and location (LOCS)
for peakIndex ← 1 to length(PKS) do
    currentPeak ← PKS[end + 1 − peakIndex]
      % Gets the peak value (reverse order)
    currentLoc ← LOCS[end + 1 − peakIndex]
      % Gets the corresponding peak location
    if currentPeak > medZ × 50 or currentPeak < medZ × 0.015 then
        z[currentLoc] ← 0
          % Removes the artifact from the signal
    end
end
```

Appendix C Supplementary Figures

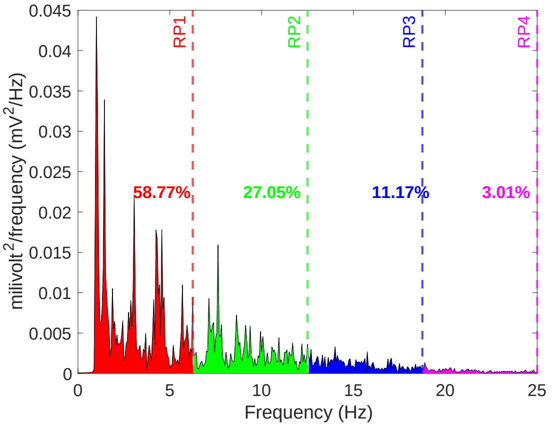

(a)

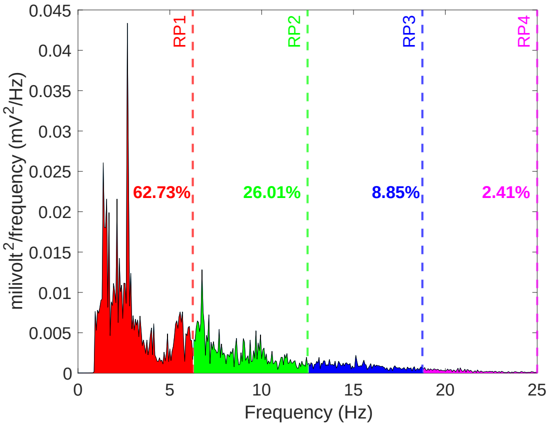

(b)

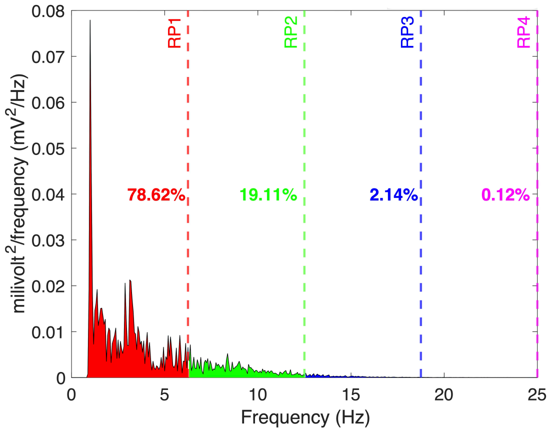

(c)

Fig. C1: PSD plot per class with the Relative Power percentage: (a) *Normal*, (b) *Moderate* and (c) *Severe*

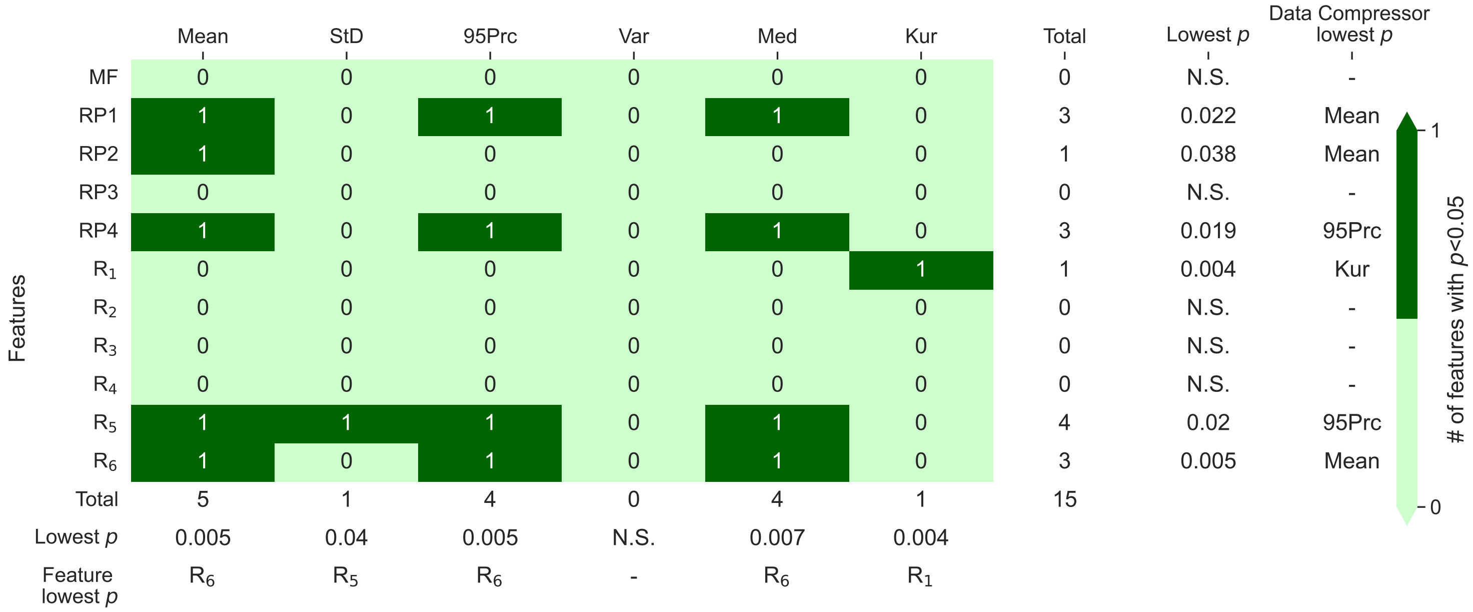

Fig. C2: ANOVA Heatmap for the "all vs. all" with the number of features per data compressor.

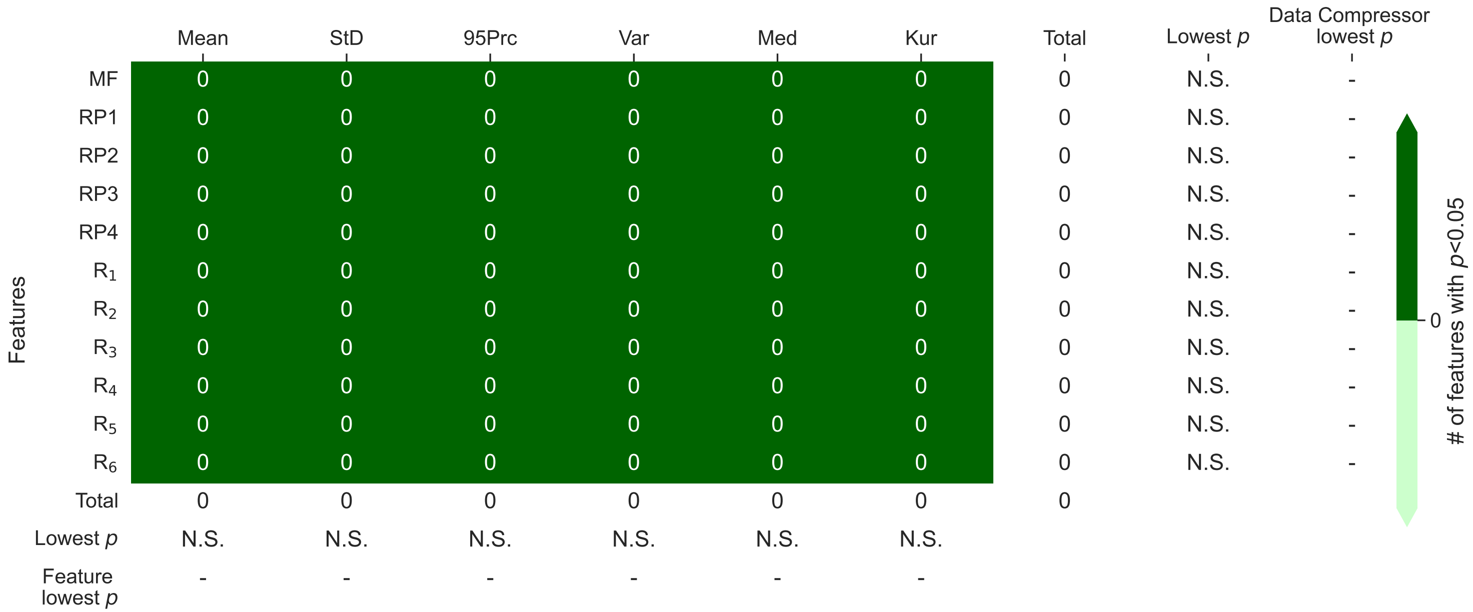

Fig. C3: T-test Heatmap for the *Normal* vs. *Moderate* with the number of significant features per data compressor.

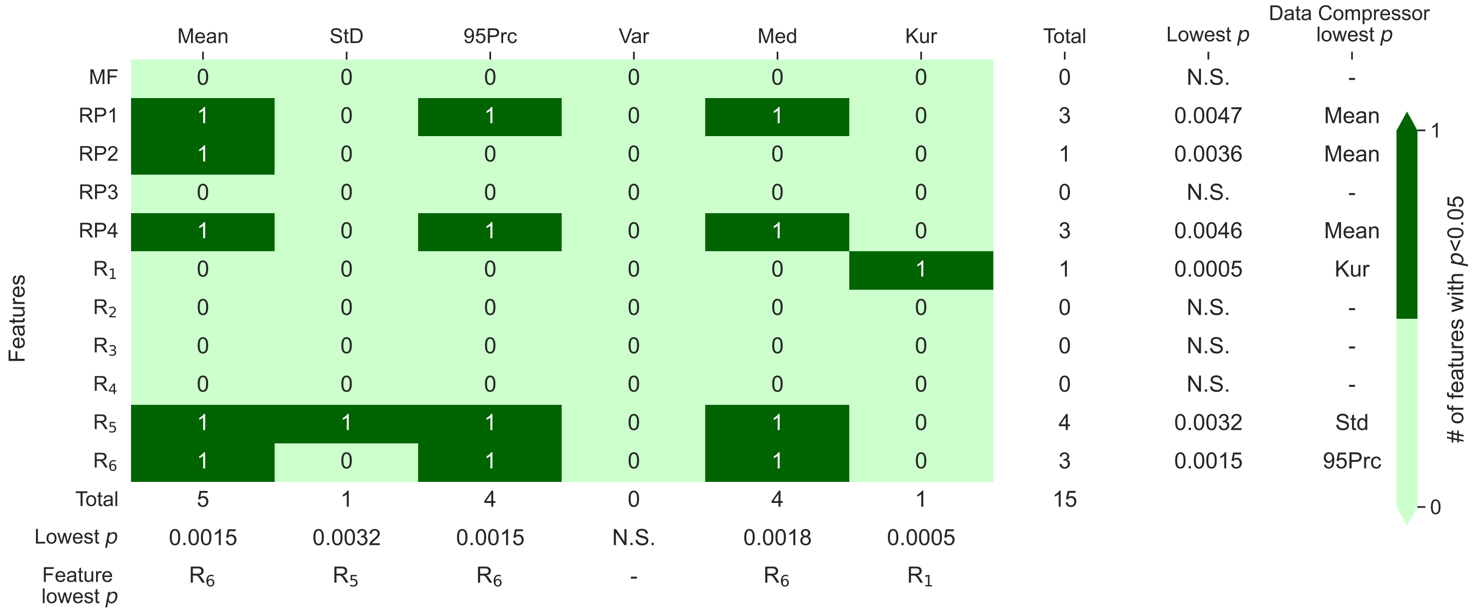

Fig. C4: T-test Heatmap for the *Normal* vs. *Severe* with the number of significant features per data compressor.

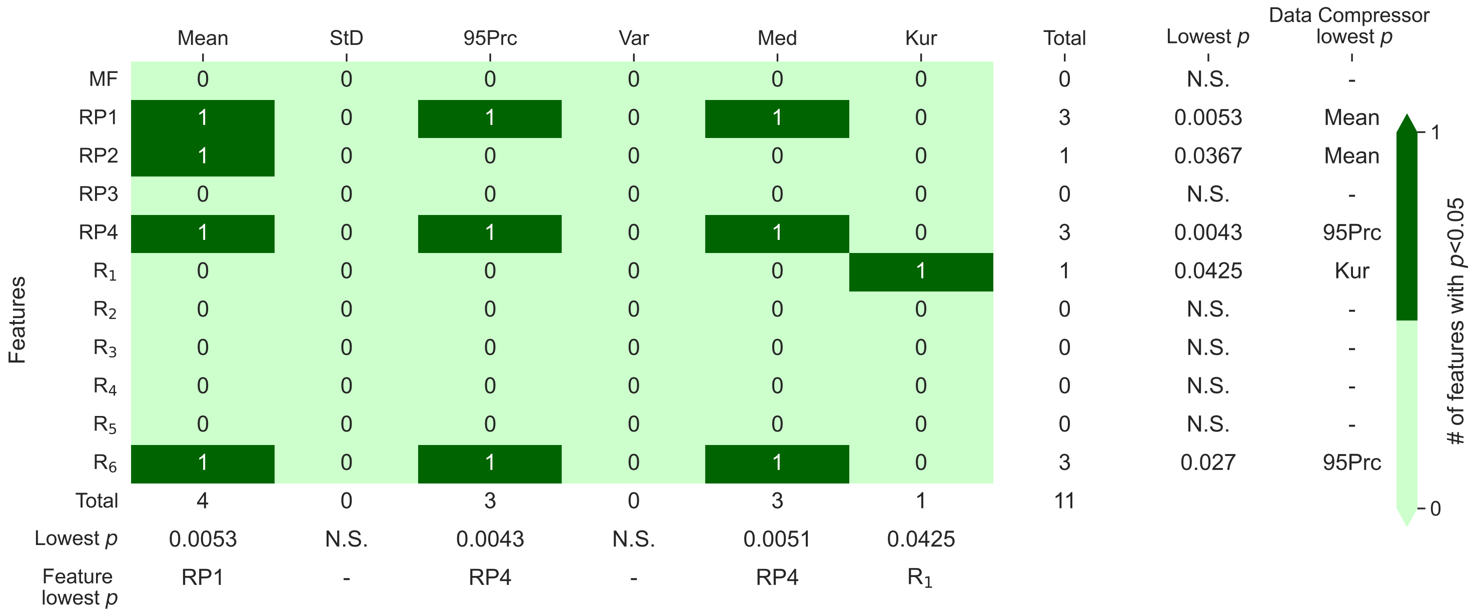

Fig. C5: T-test Heatmap for the *Moderate* vs. *Severe* with the number of significant features per data compressor.

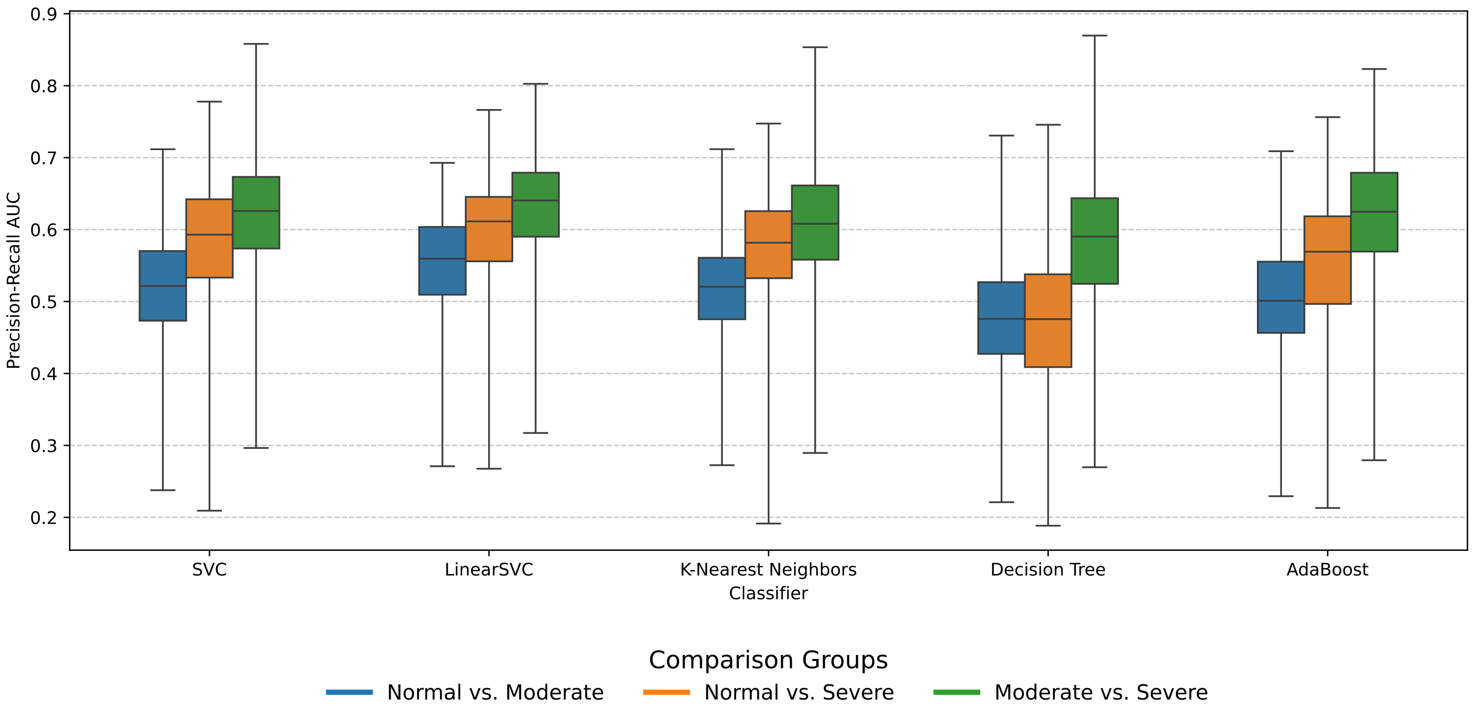

Fig. C6: Precision-Recall AUC distributions for all classifiers across the three binary comparison tasks. The tight grouping for each task highlights the robustness of the results and indicates that performance is not dependent on a single, specific classifier.

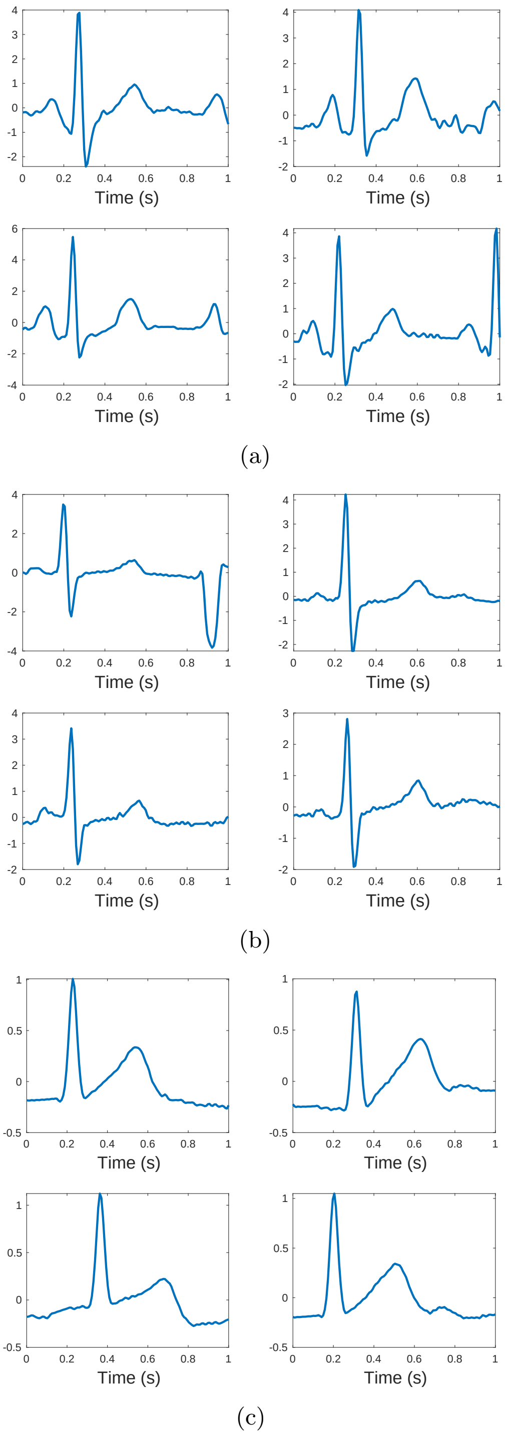

Fig. C7: ECG plot for each class: (a) *Normal*, (b) *Moderate* and (c) *Severe*
